# Isis - recovery orientated Peer Mentor Program

**DOI:** 10.1186/2050-2974-1-S1-P5

**Published:** 2013-11-14

**Authors:** Amanda Dearden, Alison Lee

**Affiliations:** 1 Isis - The Eating Issues Centre Inc, Australia

## 

This initiative is the first of this kind in Australia and supports the development of a trained volunteer mentoring network, offering opportunities for people in early recovery to develop supportive mutually beneficial mentoring relationships through regular contact with someone who has lived through and recovered from body image or eating issues for a minimum six month mentoring contract.

Recovery Mentors at Isis- The Eating Issues Centre Inc. receive Peer Mentor Training that focusses on awareness of mentoring role, boundaries, skills, risk management and self- care, developing social networks and becoming a non-professional ‘buddy’. Mentoring is provided as an additional support alongside the mentees current contact with professional medical and mental health support.

This poster will outline aims and components of the Isis Recovery Oriented Peer Mentor Program that has been trialled at Isis from late 2012 to present, and includes qualitative results and identified areas for further development. This project values lived experiences of recovery and seeks to build capacity to provide effective mentoring support, reflect on the mentoring journey and factors that contribute to recovery. Preliminary results show the program is effective in reducing social isolation, increases hope in the possibility of recovery and provides linkages back to community.

